# Perceptions of Brain Training: Public Expectations of Cognitive Benefits From Popular Activities

**DOI:** 10.3389/fnhum.2020.00015

**Published:** 2020-03-17

**Authors:** Nicole F. Ng, Robert J. Schafer, Christa M. Simone, Allen M. Osman

**Affiliations:** ^1^Department of Research and Development, Lumos Labs, San Francisco, CA, United States; ^2^Formerly of Lumos Labs, San Francisco, CA, United States

**Keywords:** cognitive training, video games, expectation, engagement, placebo effect, active control group, neuroplasticity, growth mindset

## Abstract

Many popular activities are thought by the general public to improve cognitive function. Such expectations can influence how often people engage in these activities, as well as the scientific evaluation of their putative cognitive benefits, e.g., *via* placebo effects. Here, we gathered survey data about the public’s perceptions of nine different activities commonly thought to be cognitively stimulating, including “brain-training” games. Information was collected about the degree to which participants thought each activity was beneficial for improving cognitive function and how often they engaged in each activity. The patterns of correlation between ratings reveal details about the perception of cognitive benefits and its relation to engagement. They suggest that participants varied with respect to an overarching perception of the entire set of activities, which were perceived also as divided into three clusters. Frequency of engagement and perceived cognitive benefits were positively correlated across participants for each activity considered individually. But, when the activities were compared, the magnitude of their perceived benefits was not a good predictor of their frequency of engagement (and vice versa). Though small, there were systematic demographic differences. Women were more optimistic than men about cognitive benefits. Individual participants differed in the range of their ratings of benefit across activities, and these ranges were greater for younger than older participants, suggesting that perceptions of benefit are more differentiated among the young. Besides contributing to a better understanding of public expectations of cognitive benefits, the findings of this study are relevant to the critical evaluation of such benefits. Our survey can be viewed as providing an interface between expectations held by the general public and the design of studies examining the efficacy of cognitive training. The type of information it provides could be used in the selection of activities performed by an active control group, so that control activities match the treatment intervention as closely as possible with respect to such expectations.

## Introduction

Since the mid-20th century, efforts to improve cognitive abilities involved broadly in “information processing” have become increasingly pervasive. Contemporary awareness, value, and cultivation of these abilities stem from a confluence of developments. Post-industrial economies depend on the acquisition and use of sophisticated knowledge and skills. Greater longevity in affluent societies has led to greater concern with the decline of cognitive abilities that accompanies normal and neuropathological aging. Developments in computer science and telecommunications, along with the digital revolution, have created new demands on cognitive abilities and opportunities for their cultivation. Advances in the Cognitive Sciences have increased our understanding of these abilities, and recent discoveries involving the extent of neural plasticity throughout life have provided evidence that they may be more modifiable than previously thought possible. Together, these and other developments have created a perfect storm for the cultivation of cognitive abilities.

Here, we will be concerned with some of the current and pervasive activities that are popularly thought to improve or maintain cognitive abilities. Some are long-term activities that are experienced as intellectually stimulating, such as learning a new language, playing a musical instrument, or complex games like chess or bridge. While done for many reasons, such activities are now recommended for preserving cognitive function over the course of normal healthy aging by many experts in cognitive aging. Recent examples can be found in a report sponsored by the American Association for Retired Persons (Global Council on Brain Health, 2017) and on the Mayo Clinic website (e.g., Williams, 2019). Such recommendations are based mainly on observational findings about the consequences of activities performed in the real world over long periods of time (e.g., Litwin et al., 2017; Brooker et al., 2018; Krell-Roesch et al., 2019).

Other activities involve cognitive training exercises that explicitly target specific cognitive faculties, such as selective attention, working memory, or reasoning. Cognitive training exercises have been employed as short-term interventions in the lab or clinic to enhance or preserve normal cognition or to treat disorders of cognition associated with neuropathology or pharmacological treatments (e.g., Jaeggi et al., 2008; Kesler et al., 2013; Rebok et al., 2014; D’Antonio et al., 2019). They are also available commercially to the general public, often in the form of web-based programs or apps for mobile devices, and comprise a large and growing market (SharpBrains, 2016). The efficacy of cognitive training has been studied experimentally, in many cases in randomized controlled trials, but the results so far have been mixed, and there is yet no scientific or medical consensus (National Academies of Sciences, 2017; World Health Organization, 2019). The extent to which views diverge can be seen by comparing the Consensus Letter from the Stanford Center on Longevity (2014) and Cognitive Training Data Response Letter (2014). Despite this lack of consensus, there is, however, a considerable agreement that more research is needed and that the potential benefits of cognitive training could be substantial.

Efficacy studies of cognitive training have continued to improve methodologically, both in experimental design (e.g., Simons et al., 2016) and analysis of data (e.g., Moreau et al., 2016). The work reported here is intended to contribute to this trend. Its primary concern is with a factor known to have a considerable impact on the outcomes of these studies. This factor is an individual’s expectation that performing an activity will lead to cognitive improvements. The impact of positive expectations on outcome measures in drug studies, i.e., the “Placebo Effect,” is well known. It has been well documented that participants in cognitive training studies can have similar expectations (e.g., Boot et al., 2013). And, like the placebo effect in drug studies, these expectations have been shown to influence outcome measures following a treatment unlikely to produce any real effects (Foroughi et al., 2016). It remains possible, however, that expectations, in and of themselves, can sometimes produce meaningful outcomes or influence the magnitude of genuine treatment effects. But, whatever the mechanism, to the extent that they affect outcome measures, differences in expectations of cognitive improvement might help explain some of the disparate findings in the literature.

Moving forward, it is important to control for expectancy effects in future studies. In clinical trials involving pharmaceutical interventions, placebo effects can be eliminated by “blinding” participants to whether they are in a treatment group or a control group receiving a non-active substance. Such a strategy is often impossible for behavioral interventions like cognitive training, for which membership in the treatment- or control-group may be obvious to participants. One alternative strategy would be to match the treatment and control groups for expectation of improvement, as is typically done for other non-treatment factors (e.g., demographic variables). But to do so, it would be helpful to know beforehand the expectations of participants about the treatment to be evaluated and possible alternative control activities. This was a primary motivation of the current study.

**Figure 1:**
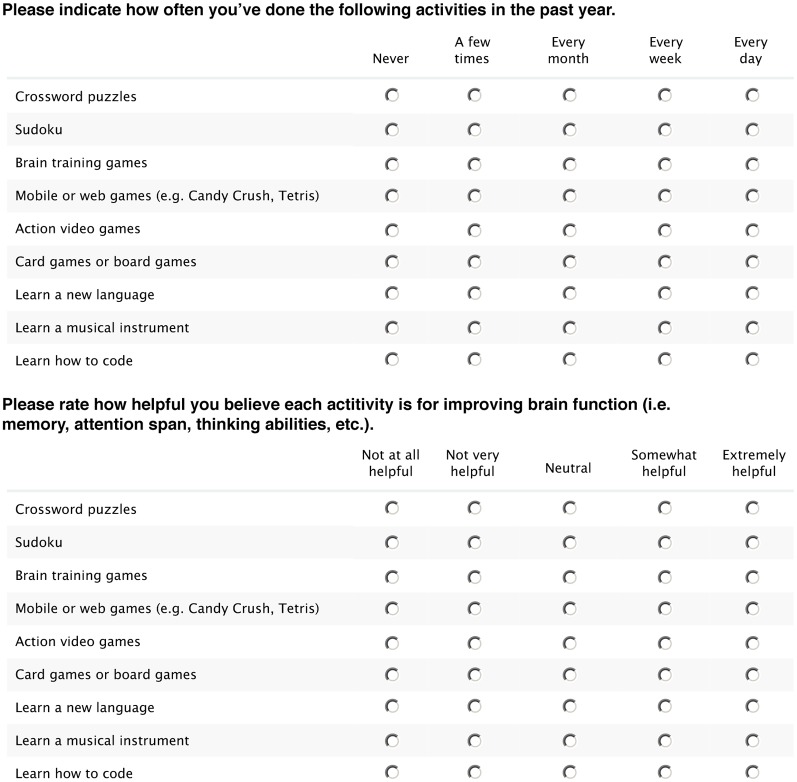
Survey questions on frequency of engagement and cognitive benefit. Shown beneath each question are the nine rated activities and labels for points on the five-point rating scales.

In this exploratory study, we gathered survey data about the public’s perceptions of nine different activities commonly thought to be cognitively stimulating, including “brain-training games.” These are shown in Figure 1 and are consistent with the definitions of cognitively stimulating activities provided in the NAS (2017) and AARP (2017) reports. Information was collected about: (1) the degree to which participants thought each activity was helpful for improving cognitive abilities; (2) how often they engaged in these activities; and (3) their age, gender, and level of educational attainment. Besides the behavioral consequences of its expected benefits, information about engagement bears on familiarity with activity and the degree to which it is intrinsically motivating. Greater motivation to engage in an activity, whether intrinsic or extrinsic, could enhance its cognitive benefits, perhaps by encouraging greater attention, effort, investment of time, or compliance in clinical interventions. Like expectations of improvement, it might also produce effects on outcome measures that need to be controlled for in efficacy studies (Motter et al., 2016).

A number of other surveys have been concerned with similar information. Some have sought to describe how the general public engages in cognitively stimulating activities. One recent survey by the AARP (Mehegan et al., 2017) examined participation and willingness to engage in such activities, as well as their association with brain health, in adults over 40. Another (Torous et al., 2016) examined the use, experience, and perceptions of smart-phone apps for brain-training among a younger tech-savvy cohort. Other recent surveys (Rabipour et al., 2018a,b) have been performed to develop psychometric instruments for comparing expectations of improvement between treatment and active control groups in efficacy studies. Our study has a different objective, which integrates the above two motivations for prior surveys. The current survey can be viewed as providing an interface between expectations held by the general public and the design of studies examining the efficacy of cognitive training. The type of information it provides could be used in the initial selection of activities performed by a control group so that they match the treatment intervention as closely as possible with respect to such expectations. Initial matching based on norms from the general public could be followed by psychometric measurement of any remaining differences between groups that occur or develop during the study. A further step might be to estimate and remove the contribution of these remaining differences from the outcome measures.

## Materials and Methods

This report concerns responses to two questions from a survey administered over the Internet in June 2015. The survey was part of a study performed with IRB approval (E&I Review Services) by Lumos Labs to investigate online neuropsychological assessments. All questions in the survey were optional, and all results from the study were anonymous and stored on secure servers.

### Recruitment

During the online recruitment process, advertisements to join an online study of cognitive tests were posted on Facebook, Google, and Craigslist. Participants provided informed consent by clicking a dialogue box on a digital consent form prior to participation in the study. To be eligible, they had to indicate that they had never taken a neuropsychological assessment, had not engaged in any cognitive training during the past year, and had never created an account on Lumosity.com. These inclusion criteria were used to minimize bias from prior assessments and cognitive training on participants’ performance on the assessments under study. Expected effect sizes in the neuropsychological assessments determined the number of participants recruited. The current study, which involves the accompanying survey, was exploratory and did not involve prior expectations about the strength of the measured relations.

### Survey

There were two main parts to the 14-question survey: a section for demographic information and a section on computer use. Included in the computer use section were two questions in which participants were asked to indicate: (1) how often they had engaged in each of nine activities during the previous year; and (2) how helpful they believed each of the nine activities to be for improving brain function related to cognition. Responses to each question about each activity were made by selecting a point on a five-point rating scale. The two questions, nine activities, and rating scales are shown in Figure 1. These responses, along with information from the demographics section on gender, age, and educational level, are analyzed in the current study.

### Participants

In our analyses, we included only responses from the 732 participants who completed all questions on the survey. The gender, age, and educational level of these participants are shown in Table 1. A majority were female (69.7%), and there was a wide range of ages (18–85 y/o, mean = 53, SD = 14.6) and educational levels (some high school to completed postgraduate degree, at least 50.4% having completed a bachelors’ or higher degree). In our demographic analyses, gender consisted of two categories (female/male). Education was divided into three categories: (1) some high school and high school diploma; (2) associate degree and some college; and (3) bachelors, masters, or professional degree, Ph.D. Age was divided into seven decades, starting at mid-teens to mid-20s and ending at mid-70s to mid-80s. There was little evidence of interaction between these three factors. No significant correlations were found between gender and educational level (−0.003, *p* > 0.05), gender and age (0.007, *p* > 0.05), or educational level and age (−0.026, *p* > 0.05).

**Table 1:** Age, gender, and educational level of survey participants.

Number of participants	732
Mean age (range, SD)	53.0 (18–85, 14.6)
Female/Male/Not declared (%)	69.7/29.5/1.0
Educational level (%)	
Some high school	4.6
High school degree (or GED)	8.9
Some college	25.4
Associate’s degree	10.1
Bachelor’s degree	25.8
Master’s degree	16.4
Professional degree	4.5
Doctoral degree (e.g., PhD)	3.7
Not declared	1.0

### Overview of Statistical Analyses

All analyses were performed on participants’ responses to the two questions about the nine activities using R statistical software (R Core Team, 2018). Since these responses consist of ratings made on a five-point scale, they are treated as ordinal measurements. Thus, except where stated otherwise, all correlations reported here are of Spearman’s rho. Demographic analyses involved non-parametric Kruskal–Wallis tests. These latter tests, when significant, are accompanied by correlations to show the direction and size of the effect. Effect sizes, in terms of the proportion of the variance, can be seen by squaring the correlations. Type I errors resulting from multiple tests was controlled by applying a criterion of 0.05 for the expected false discovery rate, which was adjusted using the Benjamini-Hochberg (BH) Procedure (Benjamini and Hochberg, 1995).

## Results

Three aspects of the data are examined in five separate sections below. First, we provide a description of how the ratings in response to each question and activity are distributed. The next two sections examine how they correlate with one another. The final two sections investigate how perceptions of cognitive benefit vary across the demographic factors.

### Rating Distributions for Perceived Benefit and Engagement

**Figure 2:**
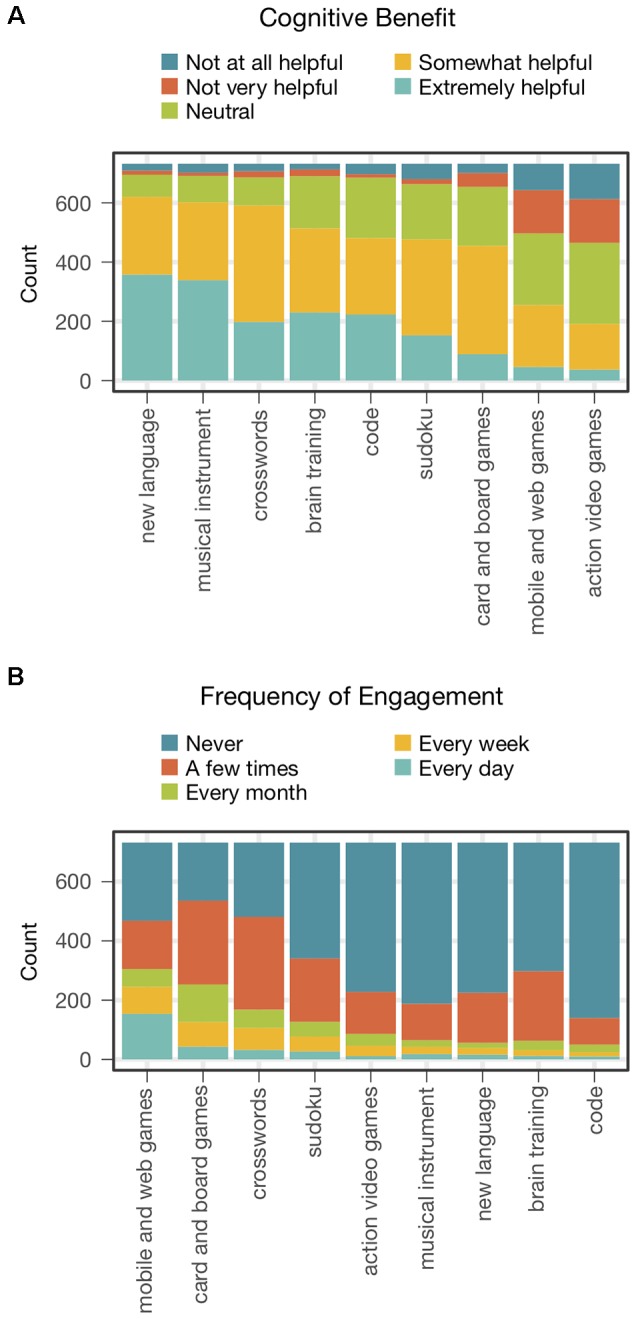
Ratings of activities on **(A)** perceived cognitive benefits and **(B)** frequency of engagement. Each panel shows stacked bar graphs of the nine activities, which are ordered by the total number of responses in the top two categories of the five-point rating scale.

Described first are the overall distributions of participants’ responses concerning the perceived benefit and frequency of engaging in each activity. The count of participants producing each rating for each activity in response to each question is shown in Figure 2. Stacked bar graphs for the “improvement” and “engagement” questions are shown respectively in Figures 2A,B. The bar graphs representing each activity are ordered within each panel by the sum of counts for the top two categories (extremely + somewhat helpful for improvement, and daily + weekly for engagement).

Participants rated complex activities, such as learning a new language or a musical instrument, as the most helpful methods for improving brain functioning. Crossword puzzles followed as the next most helpful, followed by brain training, learning to code, Sudoku, and card and board games. At the bottom of the list were casual mobile and web games, and action video games. Interestingly, the activities thought to be the most beneficial (new language, musical instrument) were among those engaged in less frequently. Conversely, activities among those thought to be the least beneficial (card and board games, mobile and web games) were engaged in the most frequently. Crossword puzzles and Sudoku were popular also among participants. Action video games were less so, and learning to code was not. In line with the inclusion criteria for the study, the level of engagement for brain training games was low.

### Correlations Between Activities in Perceived Benefit and Engagement

**Figure 3:**
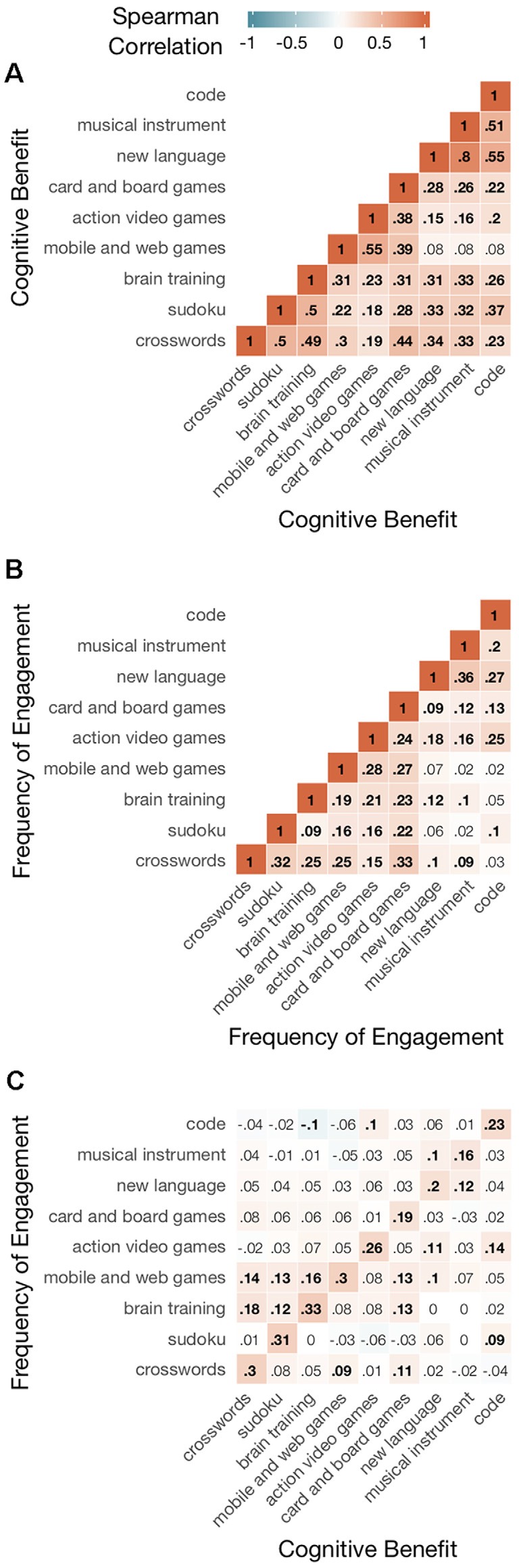
Correlations between ratings of activities on perceived cognitive benefits and frequency of engagement. Spearman’s correlations are shown, both numerically and as heat maps. Those for perceived benefit between different activities and engagement between different activities are shown respectively in Panels **(A,B)**. Panel **(C)** shows the correlations between perceived benefits and engagement, both for the same activity (main diagonal) and for two different activities (off main diagonal). Statistically significant correlations, with probabilities below a 0.05 criterion adjusted for multiple tests with the Benjamini-Hochberg Procedure, are shown in bold print.

Next, we investigated the relations between participants’ ratings of perceived benefit and engagement. Correlations across subjects are shown numerically and as heat maps in Figure 3. Red indicates positive correlations and blue indicates negative correlations. Those for perceived benefit between different activities and engagement between different activities are shown respectively in Figures 3A,B. Figure 3C shows the correlations between perceived benefits and engagement, both for the same activity (main diagonal) and for two different activities (off main diagonal). Statistically significant correlations, with probabilities below a 0.05 criterion adjusted for multiple tests, are shown in bold print. Criterion adjustment by the BH procedure was done separately for three sets of significance tests, applied respectively to the correlations in Figure 3A (36 tests), Figure 3B (36 tests), and Figure 3C (81 tests) panels.

Beliefs about improving brain function were positively correlated between activities for all possible pairs (mean = 0.318, range = 0.08–0.8, all 36 *p*’s < 0.05). This resulted in a high Cronbach’s alpha (0.81) for the nine activity ratings, which indicates that the combined set measured perceived benefit reliably (Nunnally, 1978). The high degree of intercorrelation suggests also the existence of individual differences in an overarching general perception (Cortina, 1993) of whether activities like those surveyed here can improve brain functioning.

The ratings of engagement were less correlated between activities (mean = 0.164, range = 0.02–0.36, 29 of 36 *p*’s < 0.05) and provided a less reliable measure (Cronbach’s alpha = 0.65). However, engagement in each of the nine activities did correlate significantly with the perceived benefit of the same activity (mean = 0.253, range = 0.16–0.33, all 9 *p*’s < 0.05). Interestingly, the correlation between engagement and perceived benefit was positive when considered for each activity individually, despite lower engagement in several activities thought to be more beneficial relative to those thought to be less beneficial (see Figures 2A,B).

The weakest set of correlations was between the engagement in one activity and the perceived benefit of another (mean = 0.044, range = 0–0.18, 17 of 72 *p*’s < 0.05). Moreover, there was little sign that the underlying cognitive-behavioral relations reflected by these correlations were symmetric. That is, the strength of association between engagement in activity A and perceived benefit of activity B was a poor predictor of the association between engagement in B and perception of A. To investigate such symmetry, each correlation below the main diagonal in Figure 3C was paired with the one above the diagonal involving the same two activities but switched rating scales. The correlation across these 36 pairs of correlations was not significant (0.23 for *n* = 36, *p* = 0.185).

### Similarity Between Activities in Perceived Benefit

The presence of an overarching global perception of benefit leads naturally to the question of whether it can be divided into narrower perceptual categories. In other words, were the activities perceived as belonging to different distinct groups based on their expected benefits? As can be seen in Figure 3A, the correlation in perceived benefit between different activities was not uniform across all possible pairings. Instead, we observed greater correlations within than between three different groups: (1) new language, musical instrument, coding; (2) card and board games, action video games, and web games; and (3) crossword puzzles, Sudoku, and brain training. Of the different activity types, card and board games were the most difficult to classify because of its moderate correlations with members in both groups 2 and 3.

**Figure 4:**
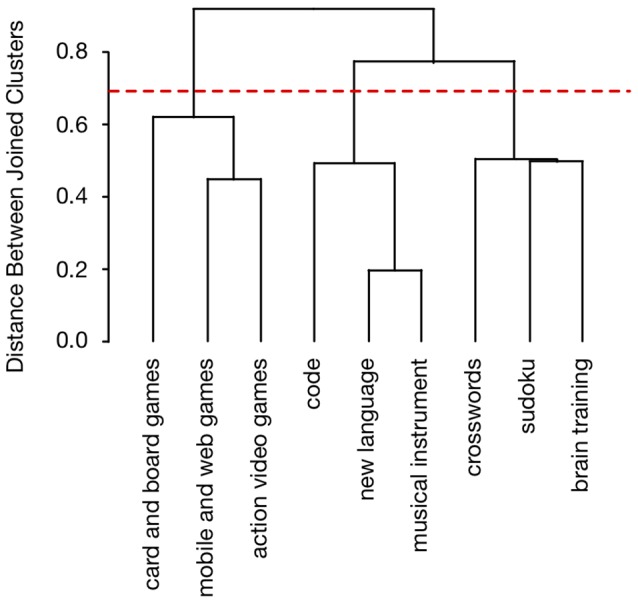
Dendrogram displaying similarity in the perceived cognitive benefits of the nine activities. The similarity of each pair of activities is based on the correlation between their respective ratings of cognitive benefits across participants. The taxonomic structure of the entire set of activities was obtained from these pairwise correlations using a hierarchical clustering algorithm (hclust function, Spearman method, R statistical package). The dashed line shows the level of the hierarchy containing the groups visually apparent in the correlation matrix (Figure 3A).

To confirm objectively the presence of these three groups of activities, we employed a hierarchical clustering algorithm, which provided a detailed representation of correlational structure based on the pairwise similarity (Euclidian distance) of the nine activities as defined by their correlation matrix (R statistical package, hclust function, Spearman method). Initially, each activity was assigned to its own cluster, and then the algorithm proceeded iteratively, at each stage joining the two most similar clusters, and continuing until there was just a single cluster. This process resulted in the dendrogram shown in Figure 4. Each bracket in this figure represents the joining of two clusters at a particular stage. The organization of activities displayed in the dendrogram at the three-cluster stage (dashed line) is the same as that observed directly from the correlation matrix (but with additional subgroupings).

### Demographic Differences in Global Perceptions of Benefit

Do the gender, educational, and age groups surveyed here differ in the extent to which they believe the nine activities benefit cognitive function? This question is addressed first for the global perception of the combined set of activities. Demographic differences in the perceived benefit of the individual activities are examined in the next section.

To characterize each participant’s overarching perception of cognitive benefit, we examined the entire distribution of their nine ratings (one for each activity). This enabled us to obtain a measure of, not only the central tendency of each participant’s ratings, but also of their range and the symmetry around their central tendency. Before obtaining these distributional parameters, the five possible responses on the rating scale were coded first as integers extending from −2 to 2, with 0 considered neutral and more positive numbers indicating a stronger belief. Next, each participant’s nine ratings were ordered from the most negative to most positive. The middle (5th) value in this ordered set corresponds to the participant’s median rating. The difference between the two extreme values (9th minus first) plus 1 was used as a measure of the range of each participant’s rating distribution. The “skew” of the distribution was measured by the difference between a subrange to the left of the median (fifth minus first value) and subrange to the right of the median (ninth minus fifth value). For skew, a positive value indicates a longer tail on the left side of the rating distribution, a negative value indicates a longer tail on the right side, and 0 indicates symmetric distribution.

**Figure 5:**
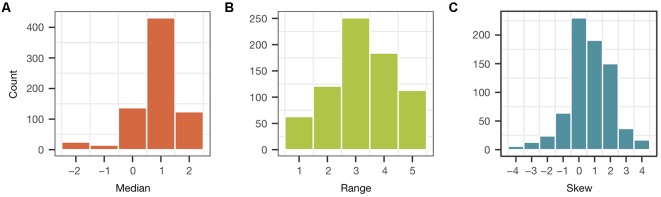
Parameters of rating distributions from individual participants. Each distribution consisted of the nine ratings of cognitive benefits, one for each activity, by a single participant. The parameters are the **(A)** median, **(B)** range, and **(C)** skew of these distributions. The histograms displayed in the figure show how these parameters, which were obtained for each individual participant, were distributed across the entire set of participants. See the text for further details.

The distribution of each (distributional) parameter across all participants is shown in Figure 5. Figure 5A shows the number of participants with medians at each possible rating value. Median ratings occurred across the entire extent of possible values. However, the central tendency of most subjects’ ratings of perceived benefit was positive. The total number of positive medians was significantly more than that of negative (555 vs. 40; *p* < 2.2 e-16) or neutral (555 vs. 137; *p* < 2.2 e-16) ones. The number of participants with each possible range (1–5) is shown in Figure 5B. A few participants had a range of 1, which means they assigned the same ratings to all activities. Those with a range of 5 used the entire set of rating values. Most participants were somewhere in between. Skew, which had possible values from −4 to 4, is shown in Figure 5C. The distribution of participants with the most negative skew had a median at the most negative rating and a tail extending to the most positive rating. Participants with the most positive skew had a distribution with a median at the most positive rating and a tail extending to the most negative. Those with a zero skew had symmetrical distributions. Most participants had symmetrical distribution or a distribution with a slightly positive (1 or 2) skew.

We turn now to an analysis of demographic differences in these distributional parameters. The demographic factors consisted of gender (0 = female, 1 = male), education (three levels), and age (seven decades). Since the parameters assumed only integer values on ordinal scales, the nonparametric Kruskal–Wallis test was employed. The test was applied nine times, once for each combination of the three parameters × three demographic factors. The *p*-values from this set of multiple tests were each compared to a 0.05 criterion adjusted using the BH procedure. When significant, tests were followed by calculation of the correlation coefficient, in order to ascertain effect size and direction. The results are shown in Table 2. Each entry contains a Kruskal–Wallis test statistic and its (unadjusted) *p*-value. Entries with significant tests (bold print) contain also a correlation coefficient.

**Table 2:** Kruskal–Wallis Tests of differences between demographic groups in distributional parameters of cognitive benefit ratings.

Parameter	Age	Gender	Education
Median	*χ*^2^(6) = 10.942, *p* = 0.09018	***χ*^2^(1) = 10.351**, *p* = 0.001294, *r*_s_ = −0.12	*χ*^2^(2) = 3.5083, *p* = 0.1731
Range	***χ*^2^(6) = 21.254**, *p* = 0.001652, *r*_s_ = −0.12	*χ*^2^(1) = 2.0271, *p* = 0.1545	*χ*^2^(2) = 0.82035, *p* = 0.6635
Skew	***χ*^2^(6) = 21.485**, *p* = 0.0015, *r*_s_ = −0.15	*χ*^2^(1) = 1.8545, *p* = 0.1733	*χ*^2^(2) = 1.0934, *p* = 0.5789

*Note. The three parameters were obtained for each participant based on the distribution of their nine cognitive benefit ratings (one for each of the nine activities). Kruskal–Wallis tests were applied to each combination of parameter × demographic. P-values were compared to a 0.05 criterion adjusted for multiple tests with the Benjamini-Hochberg Procedure. Significant tests (bold print) were followed by measurement of effect size and direction (Spearman’s rho). For Gender, females are coded 0 and males 1*.

Men and women differed significantly in the median of their ratings, with women being more positive. But no significant gender differences were found for range or skew. There were no significant differences in any of the three parameters between educational levels. Nor were there significant differences in the median between age groups. Age differences in range and skew were, however, highly significant, with younger participants showing a greater range of ratings. In other words, younger participants were less uniform (more varied) in their ratings of the different activities than older participants.

### Demographic Differences in the Perceived Benefit of Individual Activities

Do the perceived benefits of the individual activities differ across demographic groups? If so, do they differ in the same way for all activities? If different activities have different “demographic profiles,” are these profiles similar for members of the same global perceptual category (Figure 4)? To answer these questions, Kruskal–Wallis tests were performed on each combination of activity and demographic factors. Each of the 27 tests examined whether the ratings of one of the nine activities differed across one of the three demographic factors. The age, gender, and education categories were the same as in the preceding demographic analyses. Again, the *p*-value for each test was compared to a 0.05 criterion adjusted for multiple tests with the BH Procedure. Following significant tests, correlation coefficients were calculated to determine effect size and direction.

The results are shown in Table 3. Let’s first consider each demographic factor separately. For age, the tests for five of the nine activities were significant. The correlations were positive for some activities (crosswords, mobile, and web games) and negative for others (coding, musical instruments, new language). The trends in opposite directions may explain why, despite significant differences between age groups for the majority of activities, the difference in the central tendency (median) of the combined set (global perception) was not significant. For gender, tests for all but learning a new language, coding, and action video games were significant. Consistent with the significant difference between males and females in the median for global perception, all significant tests of individual activities were associated with negative correlations (males < females). For education, only the tests for crosswords, brain training, and mobile and web games were significant. Perceived benefit of each of these three activities was negatively correlated with (increased) education.

**Table 3:** Kruskal–Wallis Tests of differences between demographic groups in ratings of cognitive benefits for each activity.

Activity	Age	Gender	Education
Crosswords	***χ*^2^(6) = 16.02**, *p* = 0.0136, *r*_s_ = 0.11	***χ*^2^(1) = 6.302**, *p* = 0.0121, *r*_s_ = −0.09	***χ*^2^(2) = 19.12**, *p* = 7.04e-05, *r*_s_ = −0.16
Brain training	*χ*^2^(6) = 6.262, *p* = 0.395	***χ*^2^(1) = 8.836**, *p* = 0.00295, *r*_s_ = −0.11	***χ*^2^(2) = 13.12**, *p* = 0.00142, *r*_s_ = −0.12
Sudoku	*χ*^2^(6) = 14.001, *p* = 0.0296	***χ*^2^(1) = 6.183**, *p* = 0.0129, *r*_s_ = −0.09	*χ*^2^(2) = 2.23, *p* = 0.327
Learn new language	***χ*^2^(6) = 31.78**, *p* = 1.80e-05, *r*_s_ = −0.2	*χ*^2^(1) = 3.931, *p* = 0.0474	*χ*^2^(2) = 4.825, *p* = 0.0896
Learn to code	***χ*^2^(6) = 28.04**, *p* = 9.23e-05, *r*_s_ = −0.18	*χ*^2^(1) = 0.6952, *p* = 0.404	*χ*^2^(2) = 4.705, *p* = 0.0951
Learn musical instrument	***χ*^2^(6) = 24.19**, *p* = 0.000482, *r*_s_ = −0.17	***χ*^2^(1) = 6.153**, *p* = 0.0131, *r*_s_ = −0.09	*χ*^2^(2) = 3.866, *p* = 0.145
Card and board games	*χ*^2^(6) = 8.289, *p* = 0.218	***χ*^2^(1) = 8.208**, *p* = 0.00417, *r*_s_ = −0.11	*χ*^2^(2) = 5.452, *p* = 0.0655
Action video games	*χ*^2^(6) = 6.829, *p* = 0.337	*χ*^2^(1) = 2.387, *p* = 0.122	*χ*^2^(2) = 3.293, *p* = 0.193
Mobile and web games	***χ*^2^(6) = 37.40**, *p* = 1.47e-06, *r*_s_ = 0.15	***χ*^2^(1) = 21.69**, *p* = 3.2e-06, *r*_s_ = −0.17	***χ*^2^(2) = 10.11**, *p* = 0.00637, *r*_s_ = −0.09

*Note. Kruskal–Wallis tests were applied to the cognitive benefit ratings from each combination of activity × demographic. P-values were compared to a 0.05 criterion adjusted for multiple tests with the Benjamini-Hochberg Procedure. Significant tests (bold print) were followed by measurement of effect size and direction (Spearman’s rho). For Gender, females are coded 0 and males 1*.

Now let’s consider how similar the demographic differences were for activities within each of the three global perceptual categories (Figure 4). Learning a new language, a new musical instrument, and coding had similar demographic profiles. With one exception (gender, learning a new instrument), all three activities were identical in terms of the presence and direction of differences across all three demographic factors. The demographic profiles of crosswords, brain training, and Sudoku were less similar. Crosswords differed from brain training on one demographic (age) and from Sudoku on two (age, education). Brain training differed from Sudoku on a single demographic (education). The demographic profiles of activities in the remaining perceptual category were less similar yet. Card and board games differed from action video games on one demographic (gender) and from mobile and web games on two (age, education). The latter two activities differed on all three demographics. Similarities in profile occurred also, of course, between activities in different perceptual categories. For example, the presence and direction of all three demographic effects were the same for crosswords and mobile and web games, as well as for Sudoku and card and board games.

## Discussion

The current study highlights some of the activities that are popularly thought to improve cognitive abilities and that are often engaged in to preserve cognitive function over the course of aging. Its primary focus is on individuals’ expectations that performing these activities will lead to cognitive improvements. Expectations of improvement can have considerable impact, including placebo effects, on the outcome of studies designed to evaluate the efficacy of clinical intervention. Our survey promotes the development of an interface between expectations held by the general public and studies examining the efficacy of cognitive training. The type of information it provides can be used in the selection of activities performed by a control group so that they match the treatment intervention as closely as possible with respect to such expectations.

To illustrate, let’s consider the design of a study to test the efficacy of brain training games. As alternative forms of training for an active control group, consider crossword puzzles and action video games. Putting aside the degree to which these potential control activities might actually produce cognitive benefits, let’s focus on the degree to which they engender the expectation of such benefits. Which of the two activities is likely to produce a level of expected benefits most similar to that of brain training games in the study participants? The overall ratings of expected benefit (Figure 2), suggest that the answer is crossword puzzles. But, before accepting this conclusion, let’s consider a number of issues on which it depends and what our survey says about each.

### Perceived Cognitive Benefits

Let’s begin by looking more closely at the ratings of perceived cognitive benefit. Information beyond that provided by the average rating for each individual activity was obtained from the pattern of correlations across the entire set of activities. This pattern revealed three clusters (see dendrogram, Figure 4), wherein pairs of activities belonging to the same cluster were perceived as more similar than those belonging to different clusters. Brain training games and crossword puzzles were in the same cluster (with Sudoku), while action video games were in another cluster (with mobile and web games and card and board games).

The entire set of correlations, when considered in terms of all pairwise comparisons, was strong (high Cronbach’s alpha). Besides indicating the reliability of the overall rating averaged across activities, it suggests that our participants may have differed with respect to an overarching perception of whether activities like those surveyed can improve cognitive function. If so, this perceptual trait might be related to individual differences in belief about the malleability of cognitive abilities, which have been shown to influence learning (Dweck, 2000). Measurement of such a global perception, based on rating multiple activities, in efficacy studies might prove useful in the statistical control of expectancy effects.

### Engagement

A natural question is whether there is a relationship between engagement in and perceived benefit from the surveyed activities. Do perceived cognitive benefits influence engagement or vice versa? Given the correlational nature of our study, neither the presence nor direction of a causal connection between the two can be determined. Nonetheless, some characteristics of this relation were apparent that bear on whether the engagement can be used to predict the level of expected cognitive benefits for candidate control activities. Engagement in each activity was positively correlated with the perceived cognitive benefits of that activity. That is, participants who were more engaged in a given activity were likely to perceive it as providing greater cognitive benefits. However, the order of activities in terms of engagement is quite different than their order with respect to perceived benefits (see Figures 2A,B). Thus the level of engagement in one activity relative to another was a poor predictor of which activity was perceived to have greater cognitive benefits.

If there were individual differences in global perception of benefit that extended across the surveyed activities, they did not lead to corresponding individual differences in a general tendency to engage in these activities. Unlike perceived cognitive benefits, the level of engagement was not highly correlated across activities (low Cronbach’s alpha). That is, a participant’s level of engagement in one activity was not a good predictor of his or her level of engagement in others. Perhaps this is because one can engage in an activity for many reasons, some extrinsic and others intrinsic. Moreover, there are only so many hours in a day; Competition for a limited time may have counteracted the effects of any tendency to engage in multiple activities. In any case, the degree to which engagement correlated with itself across activities placed an upper limit on the degree to which it could correlate with perceived benefits (or anything else).

### Demographics

Ultimately, it may be possible to construct norms concerning expectations of cognitive benefits that can aid in the selection of control group activities in efficacy studies of cognitive training. An important question concerns how these norms might differ between demographic groups. The results of our exploratory study suggest that the answer is “not much.” Despite the presence of small significant effects, neither age, nor gender, nor the level of education accounted for much of the variance in ratings of expected benefit (see sizes of correlations in Tables s 2, 3). This is unlikely to be due to a lack of reliability in the ratings, given the high level of Cronbach’s alpha. Nor is it likely to be due to a lack of range in the ages (18–85) or levels of education (some high school to Ph.D.) represented in our survey sample. A minimal effect of demographics would have at least two practical advantages. One is that the norms, which would need to be based on a large number of ratings and to include many different activities, could be constructed by combining the results of many separate surveys involving different demographic groups. The other advantage is that efficacy studies involving a variety of demographic groups could make use of the same norms.

Though the effects were small, we did nevertheless find significant demographic differences. Women were more optimistic than men about the cognitive benefits of six of the nine activities. Perceived cognitive benefits increased with age for some activities and decreased for others. As a result, the general perception of benefit for the entire set of activities did not vary significantly with age, at least with respect to the median rating across all activities. Interestingly, we found that the range of ratings across the different activities was greater for younger participants, suggesting a greater differentiation between the activities with regard to perceived cognitive benefit. The perceived benefits of only three activities varied with educational level, with less optimism expressed by more educated participants in each case. A noteworthy finding is that none of the other eight activities had a pattern of demographic differences identical to that of cognitive training (Table 3). So, if serving as a control activity in an efficacy study for cognitive training, none would provide a demographic profile identical to that of the treatment.

With regard to cognitive training in particular, the present findings add to a set of conflicting results from previous surveys. Both our survey and the AARP survey (Mehegan et al., 2017) found women to be more optimistic about cognitive benefits than men. In contrast, both Rabipour and Davidson (2015) and Torous et al. (2016) reported no significant gender differences. While the AARP survey found older adults more optimistic about cognitive training, Rabipour and Davidson (2015) and Rabipour et al. (2018a) found young adults to be more optimistic. Our survey, which found no significant differences between age groups, completes the set of possibilities. Finally, while we found that perceived cognitive benefits decreased with education, Rabipour and Davidson (2015) reported that there was no relation between the two. Clearly, more research is needed to obtain an accurate picture of these demographic differences, as well as their size and any modulating factors.

### Limitations

This study should be viewed as exploratory. The survey population was small and therefore would require additional participants to build norms for future studies. Limitations in its scope include the number of activities examined and use of only two broad questions to assess respectively perceived cognitive benefits and engagement. A further limitation, which was due to the survey being embedded in a study comparing neuropsychological assessments, concerned the engagement ratings for cognitive training. To minimize bias from prior cognitive training, the inclusion criteria for the study included not having engaged in cognitive training during the past year. Despite these limitations, the study does provide the basis for initial, provisional impressions that could motivate a larger and more detailed survey or further theoretical questions concerning expectations of cognitive improvement.

What might a future survey that overcame these limitations look like? First, participants would be asked to rate more activities popularly thought to be cognitively stimulating. The AARP (2017) survey provides a good role model in this regard. Of especial value would be activities that might serve as controls in efficacy studies of cognitive training. Second, multiple types of perceived cognitive benefits and reasons for engagement would be assessed. A good example of a set of questions concerning multiple cognitive domains, such as memory, concentration, and reasoning, is the Expectation Assessment Scale developed by Rabipour and Davidson (2015) and Rabipour et al. (2018a,b). How often participants engage in an activity and the degree to which they find it intrinsically engaging (i.e., fun, enjoyable, interesting) would be assessed separately. Barriers to and concerns about engaging in different activities might be assessed also, as was done for cognitive training apps by Torous et al. (2016).

### Moving Forward

Efficacy studies of cognitive training have continued to improve methodologically. The work reported here is intended to support this trend. One of its primary concerns is with the control of effects on outcome measures due to the expectation of cognitive benefits. Such control could involve at least three approaches: (1) selection of control activities based on expectations of the general public; (2) measurement of participants’ expectations during the study; and (3) estimation and removal of the contribution of expectations to the outcome measures. The approach which our study contributes to, selection of control activities, would be useful by itself or in combination with the others. Perhaps a procedure involving all three would be optimal. Initial matching of expectations based on norms from the general public could be followed by psychometric measurement of individual differences and any remaining group differences that occur or develop during the study. A final step would be to estimate and remove the contribution of these differences from the outcome measures.

Besides being something to be controlled for, expectations might also be harnessed to improve cognitive training. Outcome measures are limited in their specificity. That is, they can be influenced by states or processes other than those they are intended to assess. In studies evaluating the efficacy of cognitive training, this would be a change in an outcome measure that is not caused by a change in a cognitive competency. Expectations of cognitive benefit are known to produce such effects (e.g., Foroughi et al., 2016). This does not imply, however, that they do not affect cognitive competencies as well. They might influence the efficacy of training (Dweck, 2000), perhaps through effects on the learning process *via* level of attention or effort or reactions to feedback. Likewise, intrinsically engaging activities might yield greater cognitive benefits beyond those due solely to better compliance or more practice. The way forward may, therefore, include studying further whether features of cognitive training thought capable of producing nonspecific effects produce specific ones as well. This will require understanding the basic mechanisms by which they influence outcome measures in efficacy studies, rather than just equating control and treatment groups or controlling statistically for their effects.

Finally, it is important to remember that the development of effective cognitive training involves more than just evaluating its efficacy in a methodologically rigorous manner. It is necessary also to identify what features and conditions lead to more effective training. Certainly, good cognitive training should be intrinsically engaging. Expectations of benefit and confidence in success may prove also to be efficacious. Surveys like ours that examine peoples’ perceptions of a wide variety of cognitively challenging activities can help us to identify features and conditions that lead to positive expectations or greater engagement, as well as others that may prove useful for the design of effective cognitive training. Given the cognitive challenges and opportunities of modern times, an interest in cognitive training seems almost inevitable. Our degree of success in this collective endeavor will have important consequences at both the level of societal benefits and individual well-being.

## Data Availability Statement

The datasets generated for this study are available on request to the corresponding author.

## Ethics Statement

The studies involving human participants were reviewed and approved by Ethical and Independent Review Services, Corte Madera, CA, USA. Written informed consent for participation was not required for this study in accordance with the national legislation and the institutional requirements.

## Author Contributions

NN and CS conceived the reported study and participated in data collection. Data analysis was performed by NN, RS, and AO. The manuscript was written by AO, NN, and RS, and edited by all the authors.

## Conflict of Interest

The study is concerned with the public perception of cognitively stimulating activities, including brain-training apps. It should therefore be mentioned that all of the authors have a current or prior professional relationship with Lumos Labs Inc., the producer of the brain-training app Lumosity. NN and RS are current employees, CS is a former employee, and AO is a paid consultant. NN and RS hold stock in the company. However, to the best of our knowledge, the outcome of the study has no commercial impact on the company or financial consequences for the authors.
